# Residual Platinum-Induced Neuropathy and the Feasibility of Second-Line Paclitaxel–Ramucirumab Therapy in Advanced Gastric Cancer: Prospective Multicenter Evidence from Japan

**DOI:** 10.3390/cancers18142243

**Published:** 2026-07-13

**Authors:** Takeshi Nagasaka, Yoshiyasu Kono, Yosuke Kito, Takayuki Ando, Yuji Negoro, Tomoyuki Abe, Hidekazu Kuramochi, Shogen Boku, Tomohiko Mannami, Junichiro Nasu, Masafumi Inoue, Masato Nakamura, Yoshihiro Okita, Yoshiaki Shindo, Takeshi Yamada, Tetsuya Maeda, Yudai Shinohara, Hiroaki Tanioka

**Affiliations:** 1 Department of Clinical Oncology, Kawasaki Medical School Hospital, Kurashiki 701-0192, Japan; tanioka@med.kawasaki-m.ac.jp; 2 Department of Advanced Oncology, Kawasaki Medical School, Kurashiki 701-0192, Japan; 3 Department of Gastroenterology and Hepatology, Faculty of Medicine, Dentistry and Pharmaceutical Sciences, Okayama University, Okayama 700-8558, Japan; ptq25yyg@okayama-u.ac.jp; 4 Department of Medical Oncology, Ishikawa Prefectural Central Hospital, Kanazawa 920-8530, Japan; kitoyo9100@gmail.com; 5 Third Department of Internal Medicine, University of Toyama, Toyama 930-0194, Japan; takayuki@med.u-toyama.ac.jp; 6 Department of Oncological Medicine, Kochi Health Sciences Center, Kochi 781-8555, Japan; tp-negoro@mx1.tiki.ne.jp; 7 Department of Gastroenterology, Steel Memorial Muroran Hospital, Muroran 050-0076, Japan; abe-tomoyuki@nshp-muroran.or.jp; 8 Department of Medical Oncology, NTT Medical Center Tokyo, Tokyo 141-8625, Japan; kuramochi.hidekazu@twmu.ac.jp; 9 Cancer Treatment Center, Kansai Medical University Hospital, Hirakata 573-1191, Japan; bokush@hirakata.kmu.ac.jp; 10 Department of Gastroenterology, NHO Okayama Medical Center, Okayama 701-1192, Japan; tmannami-gi@umin.ac.jp; 11 Department of Internal Medicine, Okayama Saiseikai General Hospital, Okayama 700-8511, Japan; jnasu@okayamasaiseikai.or.jp; 12 Department of Gastroenterology, Okayama Red Cross Hospital, Okayama 700-8607, Japan; m-inoue.er@okayama-med.jrc.or.jp; 13 Aizawa Comprehensive Cancer Center, Aizawa Hospital, Nagano 390-8510, Japan; geka-dr7@ai-hosp.or.jp; 14 Department of Cancer Center, Kagawa University Hospital, Kagawa 761-0793, Japan; yokita@med.kagawa-u.ac.jp; 15 Department of Gastroenterological Surgery, Nakadori General Hospital, Akita 010-8577, Japan; yoshiaki401223@gmail.com; 16 Department of Gastroenterological Surgery, Nippon Medical School, Tokyo 113-8603, Japan; y-tak@nms.ac.jp; 17 Department of Surgery, Konko Hospital, Asakuchi 719-0104, Japan; tetsumaeda3384@gmail.com; 18 Department of Hematology/Oncology, Japan Community Healthcare Organization Kyushu Hospital, Fukuoka 806-8501, Japan; shinohara-yudai@kyusyu.jcho.go.jp

**Keywords:** advanced gastric cancer, peripheral sensory neuropathy, oxaliplatin, paclitaxel, Ramucirumab, second-line chemotherapy

## Abstract

Background: Platinum-based doublets are standard first-line therapy for advanced gastric cancer (GC) in the Western Pacific region, but oxaliplatin frequently induces persistent peripheral sensory neuropathy (PSN). Whether residual PSN compromises the feasibility and efficacy of subsequent paclitaxel (PTX)-based therapy remains unclear. Methods: The IVY study was a prospective, multicenter observational trial conducted across 16 Japanese institutions. Patients with advanced GC progressing after fluoropyrimidine–platinum therapy received second-line PTX ± ramucirumab. Baseline PSN was assessed by CTCAE, PNQ, and FACT/GOG-Ntx. The primary endpoint was the incidence of grade ≥ 3 PSN during second-line therapy. Secondary endpoints included progression-free survival (PFS), overall survival (OS), time to treatment failure (TTF), tumor response, and patient-reported outcomes. Results: Among 156 patients (90 PSN-positive, 66 PSN-negative), grade ≥ 3 PSN occurred more frequently in the PSN-positive group (16.7% vs. 4.5%; *p* = 0.02). Median PFS was 4.0 vs. 3.9 months (*p* = 0.20), and median OS was 10.3 vs. 8.1 months (*p* = 0.04). In multivariable analysis, residual PSN was not an independent predictor of OS (HR = 0.68 for PSN-positive vs. PSN-negative, 95% CI [0.46–1.01]; *p* = 0.06). Patient-reported instruments captured greater and earlier QoL impairment in PSN-positive patients, though trajectories stabilized by 12 weeks. Conclusions: Residual PSN increases the risk of clinically relevant neurotoxicity—particularly in patients with baseline grade ≥ 2—but, in this cohort, did not appear to preclude paclitaxel-based second-line therapy when monitoring and dose modification were available. The apparent survival difference was exploratory and, after adjustment for baseline imbalance, should not be interpreted as a benefit attributable to residual PSN. Residual neuropathy should therefore prompt structured baseline assessment and early-cycle monitoring rather than routine reassurance.

## Simple Summary

Oxaliplatin, a chemotherapy drug widely used for stomach cancer, often causes lasting numbness and tingling in the hands and feet, a condition called peripheral sensory neuropathy. When the cancer progresses, patients usually switch to a second treatment based on paclitaxel, a drug that can also affect the nerves. Physicians have been concerned that giving paclitaxel to patients who already have nerve damage could worsen their symptoms, and such patients are sometimes denied this effective treatment. In this prospective study of 156 patients with advanced stomach cancer treated at 16 hospitals in Japan, we compared patients who still had nerve damage when starting second-line treatment with those who did not. Patients with pre-existing nerve damage were more likely to develop severe nerve symptoms, but this happened in only a minority, and their tumor control and survival were not worse. These findings suggest that paclitaxel-based second-line therapy can remain a reasonable option for patients with residual neuropathy, provided that nerve symptoms are carefully monitored.

## 1. Introduction

Gastric cancer (GC) remains a major global health burden. According to recent estimates from the Global Cancer Observatory and epidemiologic analyses, there were approximately 1.09 million new cases and 769,000 deaths worldwide in 2020, making GC the fifth most commonly diagnosed malignancy and the fourth leading cause of cancer-related mortality globally [1].

More recent modeling from the Global Burden of Disease (GBD) 2021 data reported 1.23 million new cases and 0.95 million deaths attributed to GC, confirming its continued ranking as one of the most lethal cancers worldwide [2]. The age-standardized incidence rate (ASIR) remains highest in East Asia, particularly Japan, Korea, and China, while mortality trends mirror these geographic disparities [3].

The practical dilemma is narrow. After oxaliplatin has injured the peripheral nerves, can a second neurotoxic agent—paclitaxel—still be given safely in the second line? Despite advances in multimodality therapy, many patients present with unresectable or recurrent disease, for which systemic chemotherapy remains the mainstay. In Japan and across Asia, standard first-line therapy typically combines a fluoropyrimidine with a platinum agent. A pivotal Phase III trial (G-SOX) demonstrated the non-inferiority of S-1 plus oxaliplatin (SOX) compared with S-1 plus cisplatin (CS), leading to the broad adoption of oxaliplatin-based regimens [4]. The most recent Japanese Gastric Cancer Treatment Guidelines (6th edition, 2021) and Pan-Asian adapted ESMO guidelines (updated) recommend oxaliplatin-containing doublets as a standard first-line option [5,6].

However, the increasing use of oxaliplatin has introduced new challenges. Chemotherapy-induced peripheral neuropathy (CIPN) is cumulative, dose-dependent, and may persist long after treatment discontinuation due to a “coasting effect.” In the Phase III G-SOX trial, sensory neuropathy was reported in 85.5% of patients receiving oxaliplatin, with grade 3 or higher events occurring in 4.7% [4]. Currently, no pharmacologic agents are recommended for prevention, and duloxetine remains the only agent with moderate evidence supporting its use for symptomatic treatment of painful CIPN [7,8]. CIPN substantially affects daily functioning, limits dose intensity, and negatively impacts quality of life (QoL).

In the second-line setting, weekly paclitaxel (PTX) has historically served as a reference regimen, having shown non-inferiority to irinotecan in the WJOG 4007 trial [9]. The Phase III RAINBOW trial subsequently established paclitaxel plus ramucirumab as a global standard, demonstrating significant survival benefit compared with paclitaxel alone [10,11]. Furthermore, the ABSOLUTE trial showed that weekly nanoparticle albumin-bound paclitaxel (nab-PTX) is non-inferior to solvent-based paclitaxel, providing an alternative formulation widely used in Japanese practice [12]. In particular, Zhang et al. reported favorable survival outcomes and acceptable safety of ramucirumab as a second-line therapy in patients with advanced or metastatic gastric or gastroesophageal adenocarcinoma, consistent with the results of pivotal trials [13].

A key clinical concern is that paclitaxel itself is neurotoxic, particularly with respect to sensory neuropathy. Given that oxaliplatin frequently induces persistent peripheral sensory neuropathy (PSN), it is clinically important to clarify whether residual oxaliplatin-induced PSN increases the risk of severe PSN during second-line PTX-based chemotherapy. Peripheral motor neuropathy (PMN), although less common, may also occur and was systematically assessed. This question is highly relevant because most pivotal PTX trials—such as the RAINBOW, ABSOLUTE, and WJOG 4007 studies—were conducted before the widespread adoption of oxaliplatin or excluded patients with pre-existing peripheral neuropathy [9,10,12]. Thus, the effect of baseline neuropathy on treatment feasibility and outcomes remains poorly defined.

Another challenge is measuring peripheral neuropathy. The most widely used clinician-reported grading system, the Common Terminology Criteria for Adverse Events (CTCAE), may underestimate the severity of neuropathy compared with patient-reported outcomes (PROs) [14,15]. Instruments such as the Patient Neurotoxicity Questionnaire (PNQ) and the Functional Assessment of Cancer Therapy/Gynecologic Oncology Group-Neurotoxicity (FACT/GOG-Ntx) have been validated and shown to capture patient experiences more sensitively [16,17]. Regulatory authorities and professional societies now encourage the integration of PROs into oncology trials to improve the accuracy of symptomatic toxicity reporting [18].

Against this backdrop, the IVY study was designed as a prospective, multicenter, observational trial in Japan to evaluate the impact of residual platinum-induced PSN on the incidence of neuropathy, treatment efficacy, and patient-reported QoL during second-line PTX-based chemotherapy for advanced GC. By incorporating both clinician- and patient-reported assessments, IVY sought to address a critical evidence gap in modern treatment sequencing, in which oxaliplatin exposure in the first line and taxane-based regimens in the second line are now standard.

## 2. Methods

### 2.1. Study Design and Patients

The IVY study was a prospective, multicenter, observational study conducted across 16 Japanese institutions between September 2018 and September 2021. The study was designed to evaluate the effect of residual platinum-induced PN on the incidence and severity of neurotoxicity, as well as on the efficacy of second-line PTX-based chemotherapy for advanced GC.

Eligible patients were required to have histologically confirmed unresectable or recurrent gastric adenocarcinoma and documented disease progression or intolerance to first-line chemotherapy that included both a fluoropyrimidine and a platinum compound (cisplatin or oxaliplatin). Additional key inclusion criteria were age ≥ 20 years, an Eastern Cooperative Oncology Group performance status (ECOG PS) of 0–2, and the presence of at least one measurable or evaluable lesion, as defined by the Response Evaluation Criteria in Solid Tumors (RECIST) version 1.1. Patients were excluded if they had a life expectancy of less than 3 months, severe or uncontrolled comorbidities (including cardiovascular disease, uncontrolled diabetes, or bleeding diathesis), active or uncontrolled infection, prior taxane exposure in any setting, or psychiatric/neurological disorders that would preclude completion of patient-reported outcome measures [19]. All patients provided written informed consent prior to enrollment.

### 2.2. Treatment

Patients received second-line chemotherapy selected at the treating physician’s discretion, in accordance with the Japanese Gastric Cancer Treatment Guidelines and the Pan-Asian-adapted ESMO Clinical Practice Guidelines. Acceptable regimens included weekly solvent-based paclitaxel (sb-PTX) ± ramucirumab, weekly nab-paclitaxel (nab-PTX) ± ramucirumab, or ramucirumab monotherapy, in line with approved practice.

Sb-PTX was administered at 80 mg/m^2^ intravenously on days 1, 8, and 15 every 28 days, with or without ramucirumab at 8 mg/kg intravenously on days 1 and 15. Nab-PTX was administered at 100 mg/m^2^ intravenously on days 1, 8, and 15 every 28 days, with or without ramucirumab, administered on the same schedule and dose. Treatment was continued until radiologic disease progression, unacceptable toxicity, physician decision, or patient withdrawal. Dose reductions, cycle delays, and supportive care interventions were permitted at the treating physician’s discretion in accordance with local institutional practice.

### 2.3. Assessments

Baseline evaluations included a complete medical history, physical examination, ECOG PS, laboratory testing, and radiologic imaging. Tumor response was assessed every 8–12 weeks using CT or MRI and evaluated per RECIST version 1.1.

Both physician-rated and patient-reported instruments were used to assess neuropathy. Physicians graded sensory (PSN) and motor (PMN) neuropathy separately using CTCAE v4.0. Patients completed the Patient Neurotoxicity Questionnaire (PNQ), which distinguishes sensory and motor symptoms [16], and the Functional Assessment of Cancer Therapy/Gynecologic Oncology Group–Neurotoxicity (FACT/GOG-Ntx) questionnaire, which evaluates neuropathy-related quality of life across 11 items on a five-point scale (score range 0–44) [17]. For this study, the primary focus was on PSN, while PMN was evaluated as an additional endpoint. Questionnaires were administered at baseline and prior to each treatment cycle.

### 2.4. Endpoints

The primary endpoint was the incidence of grade ≥ 3 PSN (CTCAE v4.0) during second-line PTX-based chemotherapy, stratified by baseline PSN status.

Secondary endpoints included: (1) overall survival (OS), defined as the time from initiation of second-line therapy to death from any cause; (2) progression-free survival (PFS), defined as the time from initiation of second-line therapy to disease progression or death from any cause; (3) time-to-treatment failure (TTF), defined as the time from treatment initiation to discontinuation for any reason, including progression, toxicity, or death; (4) objective response rate (ORR), defined as the proportion of patients achieving complete or partial response; (5) patient-reported quality of life, evaluated by PNQ and FACT/GOG-Ntx.

Safety was continuously monitored, including hematologic and non-hematologic toxicities, which were graded according to CTCAE v4.0.

### 2.5. Statistical Analysis

The target sample size of 200 patients was derived from the original IVY protocol. The final analysis population (*n* = 156) was smaller than the planned target; consequently, the study was underpowered to detect modest between-group differences in the incidence of grade ≥ 3 PSN and in survival outcomes. The primary and secondary analyses should therefore be regarded as exploratory and hypothesis-generating.

Survival distributions (PFS, OS, and TTF) were estimated using the Kaplan–Meier method and compared using the log-rank test. Hazard ratios (HRs) and 95% confidence intervals (CIs) were calculated with Cox proportional hazards models. All hazard ratios are expressed as PSN-positive vs. PSN-negative (PSN-positive as the index group and PSN-negative as the reference); thus, an HR below 1 indicates a lower hazard in the PSN-positive group. ORR and categorical outcomes were compared using Fisher’s exact test or the χ^2^ test, as appropriate. For longitudinal analyses of patient-reported outcomes (PNQ sensory, PNQ motor, and FACT/GOG-Ntx), linear mixed-effects models were fitted with fixed effects for baseline PSN status, time (as a categorical factor), and their interaction, and a random intercept for each patient. The group main effect, the time main effect, and the time × group interaction were each evaluated using likelihood-ratio tests comparing nested models estimated by maximum likelihood. As exploratory sensitivity analyses, the primary endpoint and OS were re-examined in the subgroup of patients who received oxaliplatin-based first-line chemotherapy, and the incidence of grade ≥ 3 PSN was compared between solvent-based paclitaxel and nab-paclitaxel regimens using Fisher’s exact test. Grouping was based solely on baseline clinician-graded CTCAE v4.0 sensory neuropathy (Grade 0, PSN-negative; Grade ≥ 1, PSN-positive); patient-reported instruments were used descriptively and not for grouping. To address baseline imbalance, two additional sensitivity analyses of OS were performed: a propensity-score model for baseline PSN-positive membership (age, ECOG performance status, prior oxaliplatin, first-line duration, and first-line disease control), used both for covariate adjustment and for stabilized inverse-probability weighting (IPW) [20]; and a subgroup analysis stratified by baseline CTCAE severity (Grade 1 vs. Grade ≥ 2). Concordance between clinician-reported and patient-reported neuropathy was assessed with Spearman correlation.

Multivariable Cox proportional hazards regression models were constructed to estimate the association between overall survival (OS) and potential baseline imbalances. Covariates included baseline PSN status (positive vs. negative), age (≥75 vs. <75 years), prior oxaliplatin use, duration of first-line chemotherapy (<6 vs. ≥6 months), and best response to first-line chemotherapy (disease control [complete response, partial response, or stable disease] vs. progressive disease; non-evaluable responses were excluded).

To provide an absolute measure of survival differences, we additionally estimated exploratory restricted mean survival time (RMST) [21] at pre-specified time horizons (12 and 24 months for OS; 3 and 9 months for PFS/TTF). RMST was calculated as the area under the Kaplan–Meier survival curve up to a specified time point, τ. Between-group differences (PSN-positive vs. PSN-negative) were obtained with 95% CIs using nonparametric bootstrap resampling (2000 replicates). All statistical analyses were performed using JMP, version 19.1.2 (SAS Institute Inc., Cary, NC, USA) and R version 4.5.3 (R Foundation for Statistical Computing, Vienna, Austria), with linear mixed-effects models for patient-reported outcomes fitted in R and validated in R 4.5.1 (R Foundation for Statistical Computing, Vienna, Austria) with the survival package (3.8–3) and survRM2 package (1.0–4) for the restricted mean survival time (RMST) analyses computations. A two-sided *p* < 0.05 was considered statistically significant.

### 2.6. Ethics Approval and Trial Registration

The IVY study was registered with the University Hospital Medical Information Network (UMIN) Clinical Trials Registry (UMIN000033376; registration date: 12 July 2018; public disclosure: 15 September 2018; last follow-up: 31 October 2022). The Institutional Review Board of Kawasaki Medical School approved (approval no. 3207).

### 2.7. Data Availability

The datasets generated and analyzed during the current study are not publicly available due to patient privacy restrictions, but are available from the corresponding author on reasonable request. All anonymized data supporting the findings of this study will be shared for academic purposes upon institutional approval.

## 3. Results

### 3.1. Patient Characteristics

**Figure 1:**
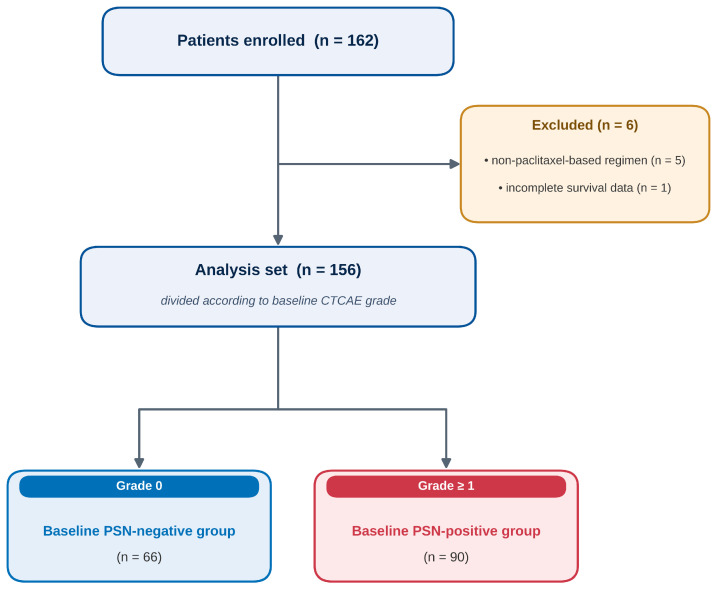
Flow diagram of patients for analysis. Peripheral sensory neuropathy (PSN) was assessed by CTCAE grading at the initiation of second-line PTX-based therapy. PSN indicates peripheral sensory neuropathy.

A total of 156 patients were included in the analysis set after excluding those who did not receive PTX-based therapy (Figure 1). Baseline characteristics of the overall study population, as well as those according to baseline PSN status at the initiation of second-line paclitaxel-based treatment, are summarized in Table 1. Of these, 90 patients (58%) were classified as baseline PSN-positive (with a baseline CTCAE grade of ≥1), and 66 patients (42%) were baseline PSN-negative (with a grade of 0). Patients in the baseline PSN-positive group were older, with a significantly higher proportion aged ≥75 years (27% vs. 12%, *p* = 0.03) and more likely to have received oxaliplatin in first-line chemotherapy (89% vs. 71%, *p* = 0.007). Importantly, patients in the baseline PSN-positive group were more likely to have achieved an objective disease control with first-line chemotherapy (82% vs. 52%, *p* = 0.0002) and to have received chemotherapy for at least 6 months (62% vs. 33%, *p* = 0.0006). Other baseline factors, including ECOG PS, tumor location, HER2 status, Lauren classification, and extent of metastatic disease, were generally balanced between the groups.

### 3.2. Primary Endpoint

The primary endpoint was the incidence of grade ≥ 3 PSN during second-line PTX-based therapy. Grade ≥ 3 PSN occurred in 15 of 90 patients (16.7%, 95% CI [9.6–26.0]) in the baseline PSN-positive group and in 3 of 66 patients (4.5%, 95% CI [0.9–12.7]) in the baseline PSN-negative group (Fisher’s exact test, *p* = 0.02; Supplementary Table S1). In the baseline PSN-positive group, most events occurred within the first two cycles, whereas such events were rare and occurred later in the baseline PSN-negative group (Supplementary Table S2).

### 3.3. Secondary Endpoints

**Figure 2:**
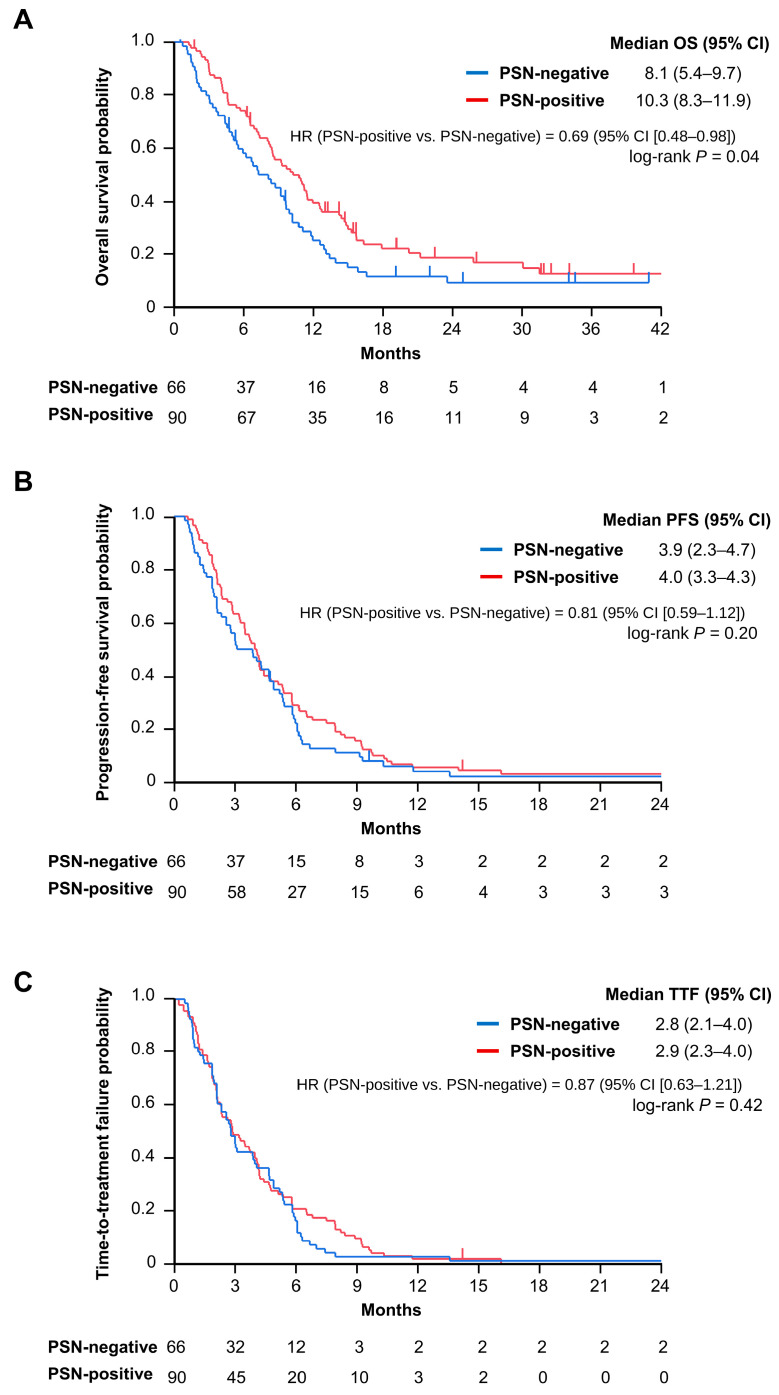
Kaplan–Meier curves for (**A**) overall survival (OS), (**B**) progression-free survival (PFS), and (**C**) time-to-treatment failure (TTF) by baseline PSN status (PSN-negative, *n* = 66; PSN-positive, *n* = 90). Curves were estimated using the Kaplan–Meier method; groups were compared using the log-rank test. Hazard ratios (HRs) and 95% confidence intervals (CIs) were obtained from Cox models. All HRs are expressed as PSN-positive vs. PSN-negative (an HR below 1 indicates a lower hazard in the PSN-positive group). Numbers at risk are shown below each panel.

For Figure 2, unadjusted HRs are presented as the baseline PSN-positive group vs. the baseline PSN-negative group. Median OS was significantly longer in the baseline PSN-positive group (10.3 months; 95% CI [8.3–11.9]) than in the baseline PSN-negative group (8.1 months; 95% CI [5.4–9.7]; HR = 0.69; 95% CI [0.48–0.98]; log-rank *p* = 0.04; Figure 2A). Median PFS was 4.0 months in the baseline PSN-positive group and 3.9 months in the baseline PSN-negative group (HR = 0.81; 95% CI [0.59–1.12]; log-rank *p* = 0.20; Figure 2B). Median TTF was 2.9 vs. 2.8 months (HR = 0.87; 95% CI [0.63–1.21]; log-rank *p* = 0.42; Figure 2C).

In the multivariable Cox model adjusting for baseline factors, PSN-positive patients showed a non-significant trend toward improved OS compared with PSN-negative patients (HR = 0.68; 95% CI [0.46–1.01]; *p* = 0.06). None of the other covariates—including age, prior oxaliplatin use, duration of first-line chemotherapy, or best first-line response—was independently associated with OS (Supplementary Table S3). The apparent OS advantage was not robust to adjustment for baseline imbalance: the unadjusted hazard ratio of 0.69 (95% CI [0.48–0.98]) attenuated to 0.78 (95% CI [0.52–1.15]) after propensity-score adjustment and to 0.85 (95% CI [0.54–1.32]; *p* = 0.46) after stabilized IPW (Supplementary Figure S1A). Subsequent (third-line) therapy was similar between groups (baseline PSN-positive 77% vs. PSN-negative 71%) [22,23], making differential post-progression treatment an unlikely explanation for the survival difference.

At 12 months, RMST was longer in PSN-positive than in PSN-negative patients (difference, +1.40 months; 95% CI [0.16–2.65]; *p* = 0.03). At 24 months, the RMST difference was +2.74 months (95% CI [0.38–5.06]; *p* = 0.03). These absolute effects were directionally consistent with the Cox model but provided a more interpretable measure of the between-group difference in restricted mean survival time. Exploratory RMST analyses for PFS and TTF at 3 and 9 months did not reveal statistically significant differences, consistent with the results from the Cox model (Supplementary Table S4).

The ORR was 23% in the baseline PSN-positive group and 19% in the baseline PSN-negative group (*p* = 0.69). The DCR was 70% and 69%, respectively. As these differences were not statistically significant, detailed results are provided in Supplementary Table S5.

The incidence of grade ≥ 3 PMN was rare, with only seven events observed in total (4.5% across the cohort). Specifically, grade ≥ 3 PMN occurred in 3 of 66 patients (4.5%, 95% CI [0.9–12.7]) in the baseline PSN-negative group and in 4 of 90 patients (4.4%, 95% CI [1.2–10.8]) in the baseline PSN-positive group, with no significant difference between the two cohorts (*p* = 1.0, Supplementary Table S6).

**Figure 3:**
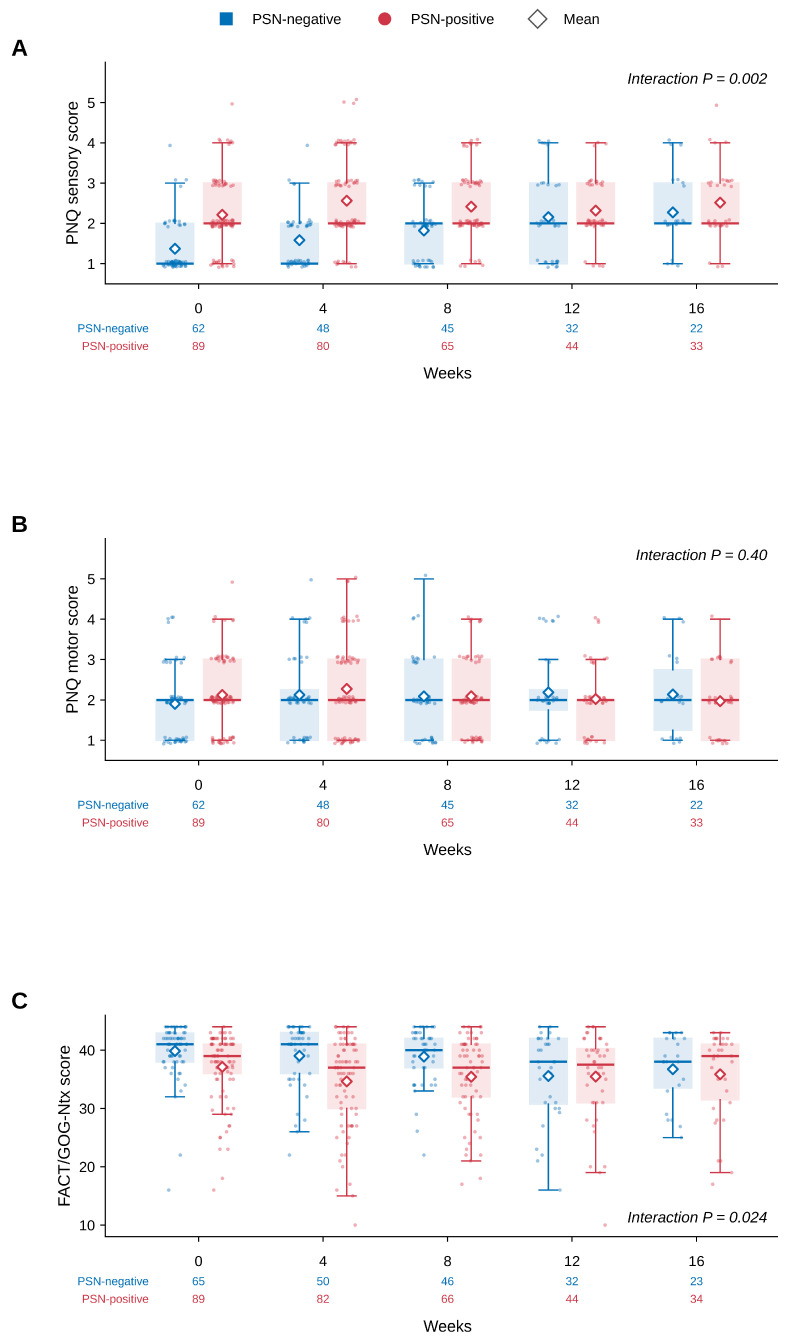
Patient-reported outcomes over time by baseline PSN status (PSN-negative, *n* = 66; PSN-positive, *n* = 90): (**A**) PNQ sensory, (**B**) PNQ motor, and (**C**) FACT/GOG-Ntx. Data are shown as box-and-whisker plots (box, interquartile range; central line, median; whiskers, 1.5× interquartile range) overlaid with individual patient values (jittered dots); open diamonds denote group means. The number of evaluable patients per group at each timepoint is shown below each panel, reflecting attrition and intermittent missing assessments. Longitudinal changes were evaluated using linear mixed-effects models with a random intercept for each patient and fixed effects for baseline PSN status, time (categorical), and their interaction; the time × group interaction *p*-values (likelihood-ratio test) were *p* = 0.002 (PNQ sensory), *p* = 0.40 (PNQ motor), and *p* = 0.024 (FACT/GOG-Ntx). Higher PNQ scores indicate worse symptoms; higher FACT/GOG-Ntx scores indicate better neurotoxicity-related QoL.

Longitudinal analyses of neuropathy-related QoL are summarized in Figure 3. For PNQ sensory scores (Figure 3A), the linear mixed-effects model showed a significantly higher overall symptom burden in patients with baseline PSN (group effect, *p* < 0.001) and a significant time × group interaction (*p* = 0.002), indicating distinct longitudinal trajectories between the two groups. In contrast, PNQ motor scores (Figure 3B) showed no significant group or interaction effect (group effect, *p* = 0.42; time × group interaction, *p* = 0.40), suggesting no meaningful group differences in motor symptoms. For FACT/GOG-Ntx scores (Figure 3C), patients with baseline PSN reported persistently lower (poorer) scores across all time points (group effect, *p* = 0.002), with a significant time × group interaction (*p* = 0.024). Detailed descriptive statistics for each assessment are provided in Supplementary Table S7.

### 3.4. Safety

The overall safety profile was consistent with previous reports of PTX-based regimens (Table 2). Grade 3 or higher hematologic toxicities included neutropenia (51% overall; 48% in PSN-negative vs. 54% in PSN-positive), anemia (9%), and thrombocytopenia (3%), with no significant differences between groups. Febrile neutropenia occurred in 7 (11%) PSN-negative and 5 (6%) PSN-positive patients (*p* = 0.36).

Among non-hematologic toxicities, grade ≥3 peripheral sensory neuropathy was significantly more frequent in the PSN-positive group (17% vs. 5%; *p* = 0.02). Fatigue was also more common in the PSN-negative group (12% vs. 2%; *p* = 0.02). Elevated AST occurred in 7 (11%) PSN-negative and 2 (2%) PSN-positive patients, reaching statistical significance (*p* = 0.04). Other grade ≥3 events—including gastrointestinal toxicities (nausea, vomiting, diarrhea, mucositis), ALT elevations, hypertension, proteinuria, and constipation—were infrequent and showed no significant differences between groups. Alopecia and malaise were common but limited to lower grades. Importantly, no unexpected toxicities were observed, supporting the feasibility of PTX-based therapy even in patients with residual neuropathy. Grade ≥ 3 PSN did not differ significantly between nab-paclitaxel and solvent-based paclitaxel regimens (8.7% [8/92] vs. 15.6% [10/64]; *p* = 0.21). In a sensitivity analysis restricted to the 127 patients who received oxaliplatin-based first-line therapy, grade ≥3 PSN remained more frequent in the PSN-positive group (18.8% vs. 4.3%; *p* = 0.03), and the longer OS in the PSN-positive group persisted (HR = 0.60; 95% CI [0.40–0.89]; *p* = 0.01; median OS 10.9 vs. 6.7 months; Supplementary Table S8). The risk of severe neurotoxicity was concentrated in patients with more advanced baseline neuropathy: grade ≥3 PSN occurred in 1 of 49 patients (2.0%) with baseline Grade 1 but 14 of 41 (34.1%) with baseline Grade ≥ 2, the latter far exceeding the 4.5% observed in PSN-negative patients (Supplementary Figure S1B and Table S9). Neurotoxicity onset was early (median cycle 1; 82% by cycle 3), and treatment was discontinued because of PSN in 9 of 90 (10.0%) PSN-positive versus 2 of 66 (3.0%) PSN-negative patients (overall 7.1%). Baseline CTCAE grade correlated moderately with patient-reported measures (PNQ sensory, Spearman ρ = 0.54; FACT/GOG-Ntx, ρ = −0.37; both *p* < 0.001; Supplementary Table S10). The number of second-line taxane cycles delivered did not differ between groups (median 3 [IQR 2–6] in PSN-positive versus 4 [IQR 2–6] in PSN-negative; *p* = 0.92), indicating comparable treatment exposure despite the higher incidence of severe neurotoxicity.

## 4. Discussion

The IVY study demonstrates that residual platinum-induced PSN increases the risk of severe neuropathy during second-line PTX-based chemotherapy but does not compromise efficacy. While OS appeared longer in the baseline PSN-positive group (10.3 vs. 8.1 months), this difference is best explained by baseline imbalances in first-line treatment history: PSN-positive patients more frequently achieved objective responses and had more prolonged oxaliplatin exposure, whereas the baseline PSN-negative group included more patients with early progression, a known adverse prognostic factor. In the multivariable Cox analysis, the apparent OS benefit was attenuated, and PSN was not confirmed as an independent prognostic factor, indicating that residual PSN primarily reflects prior treatment exposure and tumor chemosensitivity rather than exerting a direct effect on prognosis. Although baseline PSN was not an independent predictor of OS in the multivariable Cox model (HR = 0.68; *p* = 0.06; numerically favouring PSN-positive patients but not statistically significant), the exploratory RMST analysis—which does not rely on the proportional-hazards assumption—showed a significantly longer restricted mean survival time in the PSN-positive group at both 12 and 24 months (Supplementary Table S4). Because the adjusted Cox estimate was non-significant (*p* = 0.06) and the association attenuated after inverse-probability weighting, these RMST results are sensitive to model choice and should not be read as confirmatory evidence of a survival benefit. The Kaplan–Meier curves were non-parallel and diverged later in follow-up, suggesting that the proportional-hazards assumption may not fully hold and that a single hazard ratio may underestimate the late between-group difference. This pattern is most plausibly explained by greater first-line treatment sensitivity in the PSN-positive group rather than by a direct effect of residual neuropathy, and should be regarded as hypothesis-generating. The direction of causation matters here. PSN status is best interpreted as a surrogate of prior treatment exposure and response—not a biological prognostic factor—so every survival estimate in this study is non-causal and exploratory.

Notably, despite these prognostic differences, PFS and TTF during second-line therapy were similar, indicating that PTX efficacy itself was not impaired in patients with residual neuropathy. The clinical concern is real. Yet paclitaxel-based therapy remained feasible in the presence of residual neurotoxicity, provided it was actively monitored and doses were modified.

First-line therapy for advanced gastric cancer is evolving rapidly, with immune checkpoint inhibitors (CheckMate 649, KEYNOTE-062 [24,25]) and CLDN18.2- and HER2-directed agents (zolbetuximab [26,27]; trastuzumab plus pembrolizumab in KEYNOTE-811 [28,29]) extending both the duration and efficacy of first-line treatment. As a result, more patients now reach second-line therapy while still carrying residual oxaliplatin-induced neuropathy.

The phase III ARMANI trial established the timing of taxane–ramucirumab integration, showing that early switch maintenance with paclitaxel plus ramucirumab after first-line disease control improves both PFS and OS [30]. IVY complements this by defining its applicability in the increasingly common patient who reaches second-line treatment with residual oxaliplatin-induced neuropathy.

Rather than avoiding PTX out of concern for worsening neuropathy, its appropriate use with proactive monitoring and dose modification allows these patients to retain access to an effective second-line standard. Because baseline PSN-positive patients were also older, therapeutic availability should not be equated with universal suitability in older patients, in whom feasibility must be assessed individually [31].

Ultimately, our findings highlight the significance of PROs in both clinical research and clinical practice. Tools such as PNQ and FACT/GOG-Ntx consistently captured neuropathy-related QoL impairment more sensitively than clinician-reported CTCAE [14,15,18]. Routine incorporation of PROs into clinical trials and practice may enhance toxicity assessment, facilitate earlier interventions, and ultimately improve the safety and effectiveness of therapy. In our study, these differences were most pronounced early during therapy but tended to diminish by 12 weeks, suggesting that neuropathy-related QoL impairment may stabilize over time with appropriate management.

This study was underpowered relative to the original assumption (planned sample size = 200; analyzed sample size = 156), which may have limited the precision of secondary endpoints and multivariable estimates. The exclusively Japanese cohort may also influence generalizability. A further limitation is the attrition of patient-reported outcome data over time: the number of evaluable respondents declined from 62 (PSN-negative) and 89 (PSN-positive) at baseline to 22 and 33, respectively, at 16 weeks, mainly because of disease progression or treatment discontinuation. Although the linear mixed-effects models use all available data under a missing-at-random assumption, the apparent stabilization of quality-of-life scores at later time points may partly reflect the selection of patients with a more favorable prognosis who remained on treatment, and should therefore be interpreted with caution. Formal sensitivity analyses under missing-not-at-random assumptions (for example, pattern-mixture models) were not performed; the later-timepoint stabilization of patient-reported scores may thus be biased by informative dropout linked to disease progression. Additional limitations include the absence of objective neurophysiological (nerve-conduction) assessment, the lack of cumulative platinum-dose (mg/m^2^) data—for which first-line duration serves only as an indirect surrogate—and the fact that per-cycle paclitaxel dose reductions, treatment delays, relative dose intensity, supportive pharmacotherapy for neuropathy (e.g., duloxetine), and formal neuropathy-recovery trajectories were not systematically recorded—although the number of delivered taxane cycles was comparable between groups. Residual confounding may persist despite propensity-score and IPW adjustment, and the survival analyses should be regarded as exploratory.

## 5. Conclusions

IVY provides the first prospective, multicenter evidence from the Western Pacific region that residual PSN should not preclude the use of PTX-based second-line therapy (with or without ramucirumab). As GC remains a leading cause of cancer mortality in this region, our findings directly inform practice by ensuring that patients with residual neuropathy continue to access an effective and widely used second-line standard, provided proactive monitoring is implemented. At the same time, residual PSN—particularly baseline grade ≥ 2—significantly increased the risk of severe neurotoxicity and should trigger structured baseline assessment, early-cycle monitoring, patient counselling, and proactive dose modification; the survival findings are exploratory and do not indicate a benefit attributable to residual PSN. Feasibility is not uniform. It applies chiefly to patients with mild (grade 1) baseline neuropathy, whereas baseline grade ≥ 2 PSN—in whom grade ≥ 3 neurotoxicity reached 34.1%—constitutes a high-risk subgroup for whom the decision to proceed should be individualized. Second-line paclitaxel-based therapy is thus feasible with caution, not a basis for routine reassurance.

## Figures and Tables

**Table 1:** Baseline characteristics of the overall study population and according to baseline peripheral sensory neuropathy (PSN) status at initiation of second-line paclitaxel-based therapy.

Characteristic	Total	Baseline PSN-Negative	Baseline PSN-Positive	*p*-Value
	(*n* = 156), *n* (%)	(*n* = 66), *n* (%)	(*n* = 90), *n* (%)	
**Gender**				
**Male**	113 (72)	48 (73)	65 (72)	1.0 ^a^
**Female**	43 (28)	18 (27)	25 (28)	
**Age, years**				
**Median (range)**	69 (40–82)	69 (40–82)	70 (44–82)	0.26 ^b^
**<75**	124 (80)	58 (88)	66 (73)	0.03 ^a^
**≥75**	32 (20)	8 (12)	24 (27)	
**ECOG PS**				
**0**	82 (52)	32 (49)	50 (56)	0.55 ^c^
**1**	59 (38)	26 (39)	33 (37)	
**2**	15 (10)	8 (12)	7 (7)	
**Primary tumor location**				
**Upper**	49 (31)	17 (26)	32 (36)	0.42 ^c^
**Middle**	58 (38)	26 (39)	32 (36)	
**Lower**	49 (31)	23 (35)	26 (28)	
**Gastrectomy**				
**Yes**	43 (28)	20 (30)	23 (26)	0.59 ^a^
**No**	113 (72)	46 (70)	67 (74)	
**HER2 status**				
**Positive**	32 (21)	9 (14)	23 (26)	0.11 ^a^
**Negative**	122 (78)	55 (83)	67 (74)	
**Unknown**	2	2	0	
**Lauren classification**				
**Intestinal type**	74 (48)	26 (40)	48 (53)	0.11 ^a^
**Diffuse type**	81 (52)	39 (60)	42 (47)	
**Unknown**	1	1	0	
**Number of metastatic organs**				
**≤1**	81 (52)	36 (55)	45 (50)	0.63 ^a^
**≥2**	75 (48)	30 (45)	45 (50)	
**Liver metastasis**				
**Yes**	59 (38)	24 (36)	35 (39)	0.87 ^a^
**No**	97 (62)	42 (64)	55 (61)	
**Peritoneal dissemination**				
**Yes**	68 (44)	32 (48)	36 (40)	0.33 ^a^
**No**	88 (56)	34 (52)	54 (60)	
**Ascites**				
**Yes**	73 (47)	31 (47)	42 (47)	1.0 ^a^
**No**	83 (53)	35 (53)	48 (53)	
**First-line oxaliplatin use**				
**Yes**	127 (81)	47 (71)	80 (89)	0.007 ^a^
**No**	29 (19)	19 (29)	10 (11)	
**Best response to first-line chemotherapy**				
**CR**	3 (2)	0 (0)	3 (3)	0.0002 ^d^
**PR**	55 (36)	14 (21)	41 (46)	
**SD**	50 (32)	20 (31)	30 (33)	
**PD**	46 (30)	30 (47)	16 (18)	
**NE**	2	2	0	
**Duration of first-line chemotherapy**				
**<6 months**	78 (50)	44 (67)	34 (38)	0.0006 ^a^
**≥6 months**	78 (50)	22 (33)	56 (62)	
**CTCAE grade of PSN before second-line PTX**				
**0**	66 (42)	66 (100)	0 (0)	-
**1**	49 (31)	0 (0)	49 (55)	
**2**	40 (26)	0 (0)	40 (44)	
**3**	1 (1)	0 (0)	1 (1)	
**CTCAE grade of PMN before second-line PTX**				
**0**	125 (80)	64 (97)	61 (68)	0.0001 ^c^
**1**	22 (14)	2 (3)	20 (22)	
**2**	8 (5)	0 (0)	8 (9)	
**3**	1 (1)	0 (0)	1 (1)	
**Second line PTX**				
**Sb-PTX**	64 (41)	25 (38)	39 (43)	0.51 ^a^
**Nab-PTX**	92 (59)	41 (62)	51 (57)	
**Second line RAM use**				
**Yes**	134 (86)	59 (89)	75 (83)	0.35 ^a^
**No**	22 (14)	7 (11)	15 (17)	

Data are presented as *n* (%), unless otherwise indicated. *p*-values were calculated using Fisher’s exact test (a), one-way ANOVA (b), or the χ^2^ test (c), as appropriate. (d) *p*-value was calculated between CR + PR + SD and PD using Fisher’s exact test. Abbreviations: PSN, peripheral sensory neuropathy; ECOG PS, Eastern Cooperative Oncology Group performance status; PTX, paclitaxel; sb-PTX, solvent-based paclitaxel; nab-PTX, nanoparticle albumin-bound paclitaxel; RAM, ramucirumab; CR, complete response; PR, partial response; SD, stable disease; PD, progressive disease; NE, not evaluable.

**Table 2:** Treatment-related adverse events (AEs).

Adverse Event	Baseline PSN-Negative (*n* = 66)		Baseline PSN-Positive (*n* = 90)		*p*-Value
	Any Grade, *n* (%)	Grade 3–4, *n* (%)	Any Grade, *n* (%)	Grade 3–4, *n* (%)	
Febrile neutropenia	7 (11)	7 (11)	5 (6)	5 (6)	0.36
Neutropenia	48 (73)	32 (48)	72 (80)	49 (54)	0.52
Thrombocytopenia	30 (46)	3 (5)	35 (39)	2 (2)	0.65
Anemia	60 (91)	4 (6)	78 (87)	10 (11)	0.40
Increased AST	30 (46)	7 (11)	39 (43)	2 (2)	0.04
Increased ALT	16 (24)	2 (3)	25 (28)	3 (3)	1.00
Proteinuria	30 (46)	0 (0)	47 (52)	4 (4)	0.14
Peripheral sensory neuropathy	39 (59)	3 (5)	83 (92)	15 (17)	0.02
Peripheral motor neuropathy	9 (14)	3 (5)	35 (39)	4 (4)	1.00
Decreased appetite	42 (64)	4 (6)	48 (53)	7 (8)	0.76
Nausea	25 (38)	2 (3)	23 (26)	1 (1)	0.57
Vomiting	13 (20)	0 (0)	11 (12)	2 (2)	0.51
Fatigue	41 (62)	8 (12)	50 (56)	2 (2)	0.02
Malaise	38 (58)	-	49 (54)	-	-
Mucositis oral	13 (20)	1 (2)	20 (22)	1 (1)	1.00
Constipation	18 (27)	0 (0)	27 (30)	3 (3)	0.26
Diarrhea	15 (23)	0 (0)	21 (23)	0 (0)	-
Alopecia	33 (50)	-	49 (54)	-	-
Arthralgia	5 (8)	0 (0)	8 (9)	0 (0)	-
Hypertension	26 (39)	6 (9)	33 (37)	8 (9)	1.00
Epistaxis	7 (11)	0 (0)	13 (14)	0 (0)	-

Data are presented as *n* (%). *p*-values were calculated using Fisher’s exact test for comparisons of grade ≥3 events between groups. PSN, peripheral sensory neuropathy; AST, aspartate aminotransferase; ALT, alanine aminotransferase.

## Data Availability

See Data Availability Statement above.

## Supplementary Materials

The following supporting information can be downloaded at: https://www.mdpi.com/article/10.3390/cancers18142243/s1, Figure S1: Sensitivity analyses for overall survival and incidence of severe neurotoxicity by baseline neuropathy status.; Table S1: Primary endpoint and cumulative incidence of grade ≥ 3 PSN.; Tables S2: Cumulative incidence of grade ≥ 3 PSN by treatment cycle.; Table S3: Multivariable Cox proportional hazards model for overall survival.; Table S4: RMST Analysis.; Table S5: Tumor response according to baseline PSN status.; Table S6: Incidence of grade ≥ 3 peripheral motor neuropathy (PMN) by baseline PSN status.; Table S7: Patient-reported outcomes (PNQ and FACT/GOG-Ntx).; Table S8: Sensitivity analysis restricted to patients who received oxaliplatin-based first-line chemotherapy (*n* = 127).; Table S9: Grade ≥ 3 peripheral sensory neuropathy and overall survival by baseline neuropathy severity.; Table S10: Concordance between clinician-reported (CTCAE) and patient-reported neuropathy measures.
