# Supplementary material for: Residual Platinum-Induced Neuropathy and the Feasibility of Second-Line Paclitaxel–Ramucirumab Therapy in Advanced Gastric Cancer: Prospective Multicenter Evidence from Japan
Source: Cancers (Basel). 2026 Jul 13;18(14):2243. doi: 10.3390/cancers18142243 (PMC13406993; doi:10.3390/cancers18142243)
Supplement: Supplementary file 1 [file cancers-18-02243-s001.zip › cancers-4410231-supplementary.pdf]

Supplementary Files

Residual Platinum-Induced Neuropathy and the Feasibility of Second-Line Paclitaxel–Ramucirumab Therapy in Advanced Gastric Cancer: Prospective Multicenter Evidence from Japan

Takeshi Nagasaka[1#], Yoshiyasu Kono[2], Yosuke Kito[3], Takayuki Ando[4], Yuji Negoro[5], Tomoyuki Abe[6], Hidekazu Kuramochi[7], Shogen Boku[8], Tomohiko Mannami[9], Junichiro Nasu[10], Masafumi Inoue[11], Masato Nakamura[12], Yoshihiro Okita[13], Yoshiaki Shindo[14], Takeshi Yamada[15], Tetsuya Maeda[16], Yudai Shinohara[17], and Hiroaki Tanioka[1]

1. Department of Clinical Oncology, Kawasaki Medical School Hospital, Kurashiki, Japan
2. Department of Gastroenterology and Hepatology, Faculty of Medicine, Dentistry and Pharmaceutical Sciences, Okayama University, Okayama, Japan
3. Department of Medical Oncology, Ishikawa Prefectural Central Hospital, Ishikawa, Japan
4. Third Department of Internal Medicine, University of Toyama, Toyama, Japan
5. Department of Oncological Medicine, Kochi Health Sciences Center, Kochi, Japan
6. Department of Gastroenterology, Steel Memorial Muroran Hospital, Muroran, Japan
7. Department of Medical Oncology, NTT Medical Center Tokyo, Tokyo, Japan
8. Cancer Treatment Center, Kansai Medical University Hospital, Hirakata, Japan
9. Department of Gastroenterology, NHO Okayama Medical Center, Okayama, Japan
10. Department of Internal Medicine, Okayama Saiseikai General Hospital, Okayama, Japan
11. Department of Gastroenterology, Okayama Red Cross Hospital, Okayama, Japan
12. Aizawa Comprehensive Cancer Center, Aizawa Hospital, Nagano, Japan
13. Department of Cancer Center, Kagawa University Hospital, Kagawa, Japan.
14. Department of Gastroenterological Surgery, Nakadori General Hospital, Akita, Japan.
15. Department of Gastroenterological Surgery, Nippon Medical School, Tokyo, Japan.
16. Department of Surgery, Konko Hospital, Asakuchi, Japan
17. Department of Hematology/Oncology, Japan Community Healthcare Organization Kyushu Hospital, Fukuoka, Japan

Supplementary Figure S1.

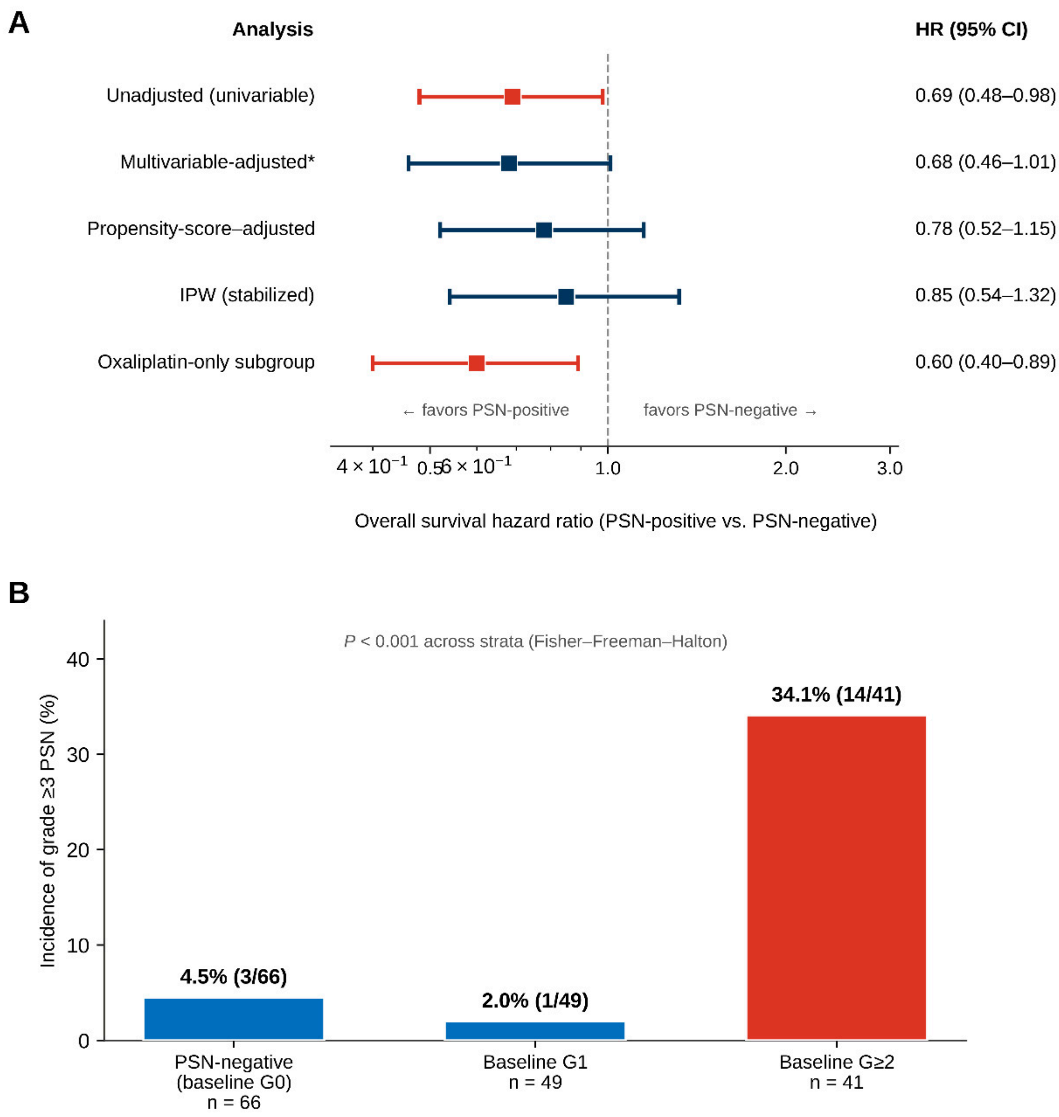

Supplementary Figure S1. Sensitivity analyses for overall survival and incidence of severe neurotoxicity by baseline neuropathy status. (A) Forest plot of OS hazard ratios (PSN-positive vs PSN-negative) across unadjusted, multivariable, propensity-score–adjusted, IPW, and oxaliplatin-only analyses; (B) grade ≥3 PSN incidence by baseline severity.

Supplementary Table S1. Primary endpoint and cumulative incidence of grade ≥3 PSN.

Group	Events (≥3 PSN)	Incidence, % (95% CI)	*p*-Value
**Baseline PSN-negative (*n* = 66)**	3	4.5 (0.9–12.7)	0.02
**Baseline PSN-positive (*n* = 90)**	15	16.7 (9.6–26.0)	

Incidence rates with 95% confidence intervals (CI) are shown. *p*-value was calculated by Fisher's exact test. PSN, peripheral sensory neuropathy.

Supplementary Table S2. Cumulative incidence of grade ≥3 PSN by treatment cycle.

Cycle	Events PSN–	Events PSN+	Cumulative PSN–	Cumulative PSN+	CumRate PSN– (%)	CumRate PSN+ (%)
1	0	6	0	6	0	6.7
2	1	5	1	11	1.5	12.2
3	2	2	3	13	4.5	14.4
4	0	1	3	14	4.5	15.6
5	0	1	3	15	4.5	16.7
6	0	0	3	15	4.5	16.7
7	0	0	3	15	4.5	16.7
8	0	0	3	15	4.5	16.7
9	0	0	3	15	4.5	16.7

Data are presented as the number of patients (*n*). "Events" indicate new onset of grade ≥3 peripheral sensory neuropathy (PSN) during each treatment cycle. "Cumulative" represents the total number of patients who had experienced grade ≥3 PSN up to that cycle. "CumRate" denotes cumulative incidence rates, calculated as the proportion of patients with grade ≥3 PSN among all patients in each group. PSN–, patients without baseline PSN (CTCAE grade 0); PSN+, patients with baseline PSN (CTCAE grade ≥1). Percentages are shown relative to the total number of patients in each baseline PSN group.

Supplementary Table S3. Multivariable Cox proportional hazards model for overall survival.

Variable	HR	95% CI Lower	95% CI Upper	*p*-Value
Baseline PSN status (Positive vs. Negative)	0.68	0.46	1.01	0.06
Age (≥75 vs. <75)	0.95	0.61	1.48	0.81
Oxaliplatin usage in 1st line (Yes vs. No)	1.22	0.75	1.99	0.42
Duration of first-line chemotherapy (≥6 months vs. <6 months)	0.87	0.56	1.36	0.53
First-line response (CR + PR + SD vs. PD)	0.81	0.51	1.29	0.37

HRs were adjusted for all listed covariates. Comparisons are coded as shown in the 'Variable' column; HR < 1 favors the first-listed level (PSN-positive; age ≥75; oxaliplatin use; duration ≥6 months; CR + PR+SD). CI denotes confidence interval.

Supplementary Table S4. RMST Analysis.

Endpoint	τ (months)	RMST difference (PSN+ – PSN–)	95% CI	*p*-Value
OS	12	+1.40	0.16 to 2.65	0.03
	24	+2.74	0.38 to 5.06	0.03
PFS	3	+0.21	−0.03 to 0.45	0.08
	9	+0.60	−0.26 to 1.46	0.16
TTF	3	+0.02	−0.26 to 0.30	0.90
	9	+0.34	−0.47 to 1.18	0.43

RMST for OS was computed as the area under the Kaplan–Meier curve up to the time point τ. Differences and 95% CIs were estimated via nonparametric bootstrap with 2,000 replicates. Group medians and their 95% CIs were obtained via bootstrap (2,000 replicates). Positive RMST differences favor PSN-positive. Exploratory RMST analyses for PFS and TTF did not show statistically significant differences at either $\tau = 3$ or $\tau = 9$ months, consistent with the adjusted Cox model.

Supplementary Table S5. Tumor response according to baseline PSN status.

Response	All (*n* = 148)	Baseline PSN-negative group (*n* = 64)	Baseline PSN-positive group (*n* = 84)
**CR, n (%)**	1 (0.7)	0 (0)	1 (1.2)
**PR, n (%)**	30 (20.0)	12 (18.8)	18 (21.4)
**SD, n (%)**	72 (48.6)	32 (50.0)	40 (47.6)
**PD, n (%)**	42 (28.4)	18 (28.1)	24 (28.6)
**NE, n (%)**	3 (2.0)	2 (3.1)	1 (1.2)
**ORR, % (95% CI)**	20.9 (15.2–28.2)	18.8 (11.1–30.0)	22.6 (15.0-32.6)
**DCR, % (95% CI)**	69.6 (61.8–76.4)	68.8 (56.6–78.8)	70.2 (59.8-79.0)

Data are presented as n (%). Response was evaluated according to RECIST version 1.1. ORR (objective response rate) was defined as the proportion of patients achieving complete response (CR) or partial response (PR). DCR (disease control rate) was defined as the proportion of patients achieving CR, PR, or stable disease (SD). 95% confidence intervals (CI) were calculated using the Wilson score method. PSN, peripheral sensory neuropathy; NE, not evaluable.

Supplementary Table S6. Incidence of grade ≥3 peripheral motor neuropathy (PMN) by baseline PSN status.

Group	Events (≥3 PMN)	Incidence (%)	*p*-Value
**Baseline PSN-negative (*n* = 66)**	3	4.5 (95% CI 0.9–12.7)	1.0
**Baseline PSN-positive (*n* = 90)**	4	4.4 (95% CI 1.2–10.8)	

Data are presented as *n* (%). Incidence rates with 95% confidence intervals (CI) are shown. *P*-values were calculated using Fisher's exact test. PSN, peripheral sensory neuropathy; PMN, peripheral motor neuropathy.

Supplementary Table S7. Patient-reported outcomes (PNQ and FACT/GOG-Ntx).

	Baseline	4 weeks	8 weeks	12 weeks	16 weeks
**PNQ sensory, mean (SD), analyzed no.**					
Baseline PSN-negative	1.37 (0.66), 62	1.58 (0.71), 48	1.82 (0.75), 45	2.16 (1.08), 32	2.27 (0.94), 22
Baseline PSN-positive	2.21 (0.89), 89	2.56 (1.03), 80	2.42 (0.85), 65	2.32 (0.74), 44	2.52 (0.91), 33
**PNQ motor, mean (SD), analyzed no.**					
Baseline PSN-negative	1.90 (0.86), 62	2.12 (1.00), 48	2.09 (1.00), 45	2.19 (1.03), 32	2.14 (0.99), 22
Baseline PSN-positive	2.12 (0.88), 89	2.27 (1.11), 80	2.09 (0.91), 65	2.02 (0.85), 44	1.97 (0.85), 33
**FACT/GOG-Ntx, mean (SD), analyzed no.**					
Baseline PSN-negative	39.84 (4.75), 65	39.02 (5.35), 50	38.90 (4.91), 46	35.57 (7.57), 32	36.73 (5.82), 23
Baseline PSN-positive	37.13 (5.92), 89	34.63 (7.79), 82	35.43 (7.04), 66	35.47 (7.65), 44	35.85 (7.58), 34

p-values were derived from linear mixed-effects models with a random intercept for each patient and fixed effects for baseline PSN status, time (categorical), and their interaction (likelihood ratio tests). Significant time × group interactions were observed for PNQ sensory ($p = 0.002$) and FACT/GOG-Ntx ($p = 0.024$), whereas PNQ motor showed no significant group or interaction effect ($p = 0.42$ and $p = 0.40$, respectively).

Supplementary Table S8. Sensitivity analysis restricted to patients who received oxaliplatin-based first-line chemotherapy (*n* = 127).

Outcome	PSN-negative (*n* = 47)	PSN-positive (*n* = 80)	*p*-Value
**Grade ≥3 PSN, *n* (%)**	2 (4.3)	15 (18.8)	0.03
**Median OS, months**	6.7	10.9	0.01

Analysis restricted to the 127 patients who received oxaliplatin-based first-line chemotherapy. The overall survival hazard ratio (PSN-positive vs. PSN-negative) was 0.60 (95% CI 0.40–0.89). *p*-values were calculated by Fisher's exact test (grade ≥3 PSN) and the log-rank test (median OS).

Supplementary Table S9. Grade ≥3 peripheral sensory neuropathy and overall survival by baseline neuropathy severity.

Baseline severity	*n*	Grade ≥3 PSN, *n* (%)	Median OS, months
**PSN-negative (Grade 0)**	66	3 (4.5)	8.1
**Baseline Grade 1**	49	1 (2.0)	10.0
**Baseline Grade ≥2**	41	14 (34.1)	11.0

Grouping by baseline CTCAE v5.0 sensory grade. The risk of severe neurotoxicity was concentrated in the baseline Grade ≥2 stratum (Fisher–Freeman–Halton $p < 0.001$ across strata). Neurotoxicity onset was early (median cycle 1; 82% by cycle 3). Survival analyses are exploratory.

Supplementary Table S10. Concordance between clinician-reported (CTCAE) and patient-reported neuropathy measures.

Comparison	Spearman *ρ*	*p*-Value	*n*
**Baseline CTCAE vs. PNQ sensory**	0.54	< 0.001	151
**Baseline CTCAE vs. FACT/GOG-Ntx**	−0.37	< 0.001	154

A negative correlation with FACT/GOG-Ntx is expected because higher FACT/GOG-Ntx scores indicate better function (fewer symptoms). Clinician- and patient-reported measures were concordant in direction and moderately correlated in magnitude.
